# Supplementary material for: Impact of between-tissue differences on pan-cancer predictions of drug sensitivity
Source: PLoS Comput Biol. 2021 Feb 25;17(2):e1008720. doi: 10.1371/journal.pcbi.1008720 (PMC7906305; doi:10.1371/journal.pcbi.1008720)
Supplement: S1 Text — Fig A. Within- and between-tissue RNA expression similarity. (A, B) Scatterplots of the first two dimensions from principal component (PC) analysis on transcriptome data. Each point represents a cell line and is colored by dataset (A) and tissue-of-origin (B). (C,D,E) Heatmaps of within- and between-tissue RNA expression similarity. Heatmap colors indicate the mean ranked RNA expression correlation (Spearman’s ρ) across genes with highly-variable expression (see Methods) for 100 randomly-selected pairs of cell lines of the indicated tissues. Black outlines indicate comparisons within the same tissue type. Expression correlation is shown for cell lines (C) within Klijn 2015, (D) within CCLE, and (E) between the two datasets. Fig B. Evaluating thresholds to classify cell lines as sensitive or resistant to four MEK inhibitor screens. (A-D) IC50 distributions for the four MEKi screens in Klijn 2015 (A, B) and CCLE 2019 (C, D). Vertical dotted lines: 1 μM threshold used to classify cell lines as sensitive or resistant to MEK inhibition. Vertical dashed line in (B): 6 μM threshold tested for GDC-0973 in Klijn 2015. (E,F) Area under the receiver operating characteristic (auROC) performances calculated by comparing observed MEKi response (x-axis) with predicted MEKi response from fK2 models using 1 μM (dark gray bars) or 6 μM (light gray) thresholds trained with logistic regression (E) or random forest (F) algorithms. Fig C. Prediction performances as assessed in the full pan-cancer cell line set (left-most column) and within 10 cancer types (the next 10 columns). Heatmaps indicate rank correlation (Spearman’s ρ) between observed and predicted MEKi responses (Fig 2) based on regularized (top) and logistic (bottom) regression prediction models. Each row is for a specific combination of training data and test data, over two MEK inhibitors and two datasets. Also shown are the mean performance for each column (bottom row). Dendrogram at the top depicts hierarchical clustering o [file pcbi.1008720.s005.docx]

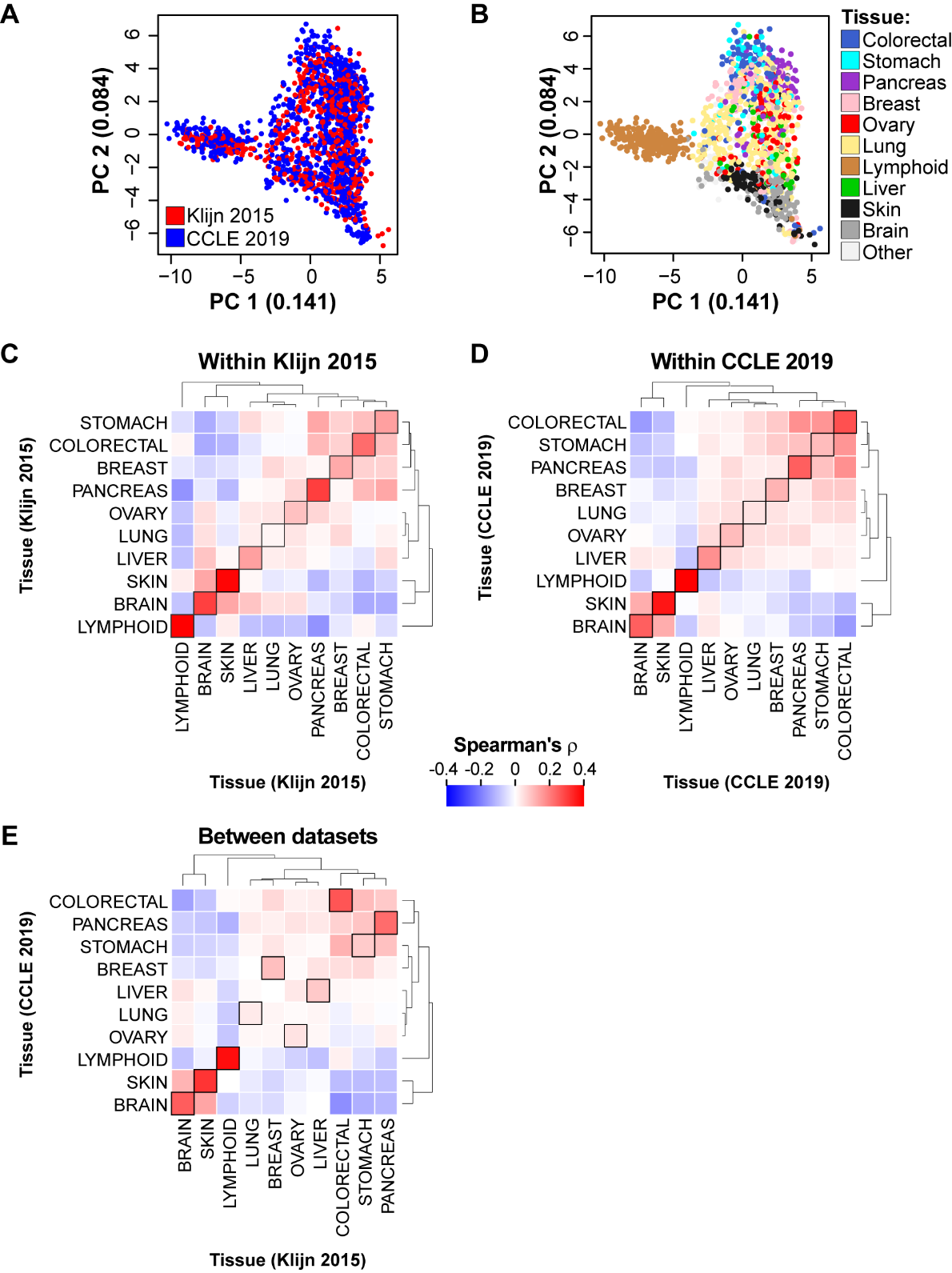


**Figure A.** Within- and between-tissue RNA expression similarity. **(A, B)** Scatterplots of the first two dimensions from principal component (PC) analysis on transcriptome data. Each point represents a cell line and is colored by dataset **(A)** and tissue-of-origin **(B)**. **(C,D,E)** Heatmaps of within- and between-tissue RNA expression similarity. Heatmap colors indicate the mean ranked RNA expression correlation (Spearman’s ρ) across genes with highly-variable expression (see Methods) for 100 randomly-selected pairs of cell lines of the indicated tissues. Black outlines indicate comparisons within the same tissue type. Expression correlation is shown for cell lines **(C)** within Klijn 2015, **(D)** within CCLE, and **(E)** between the two datasets.


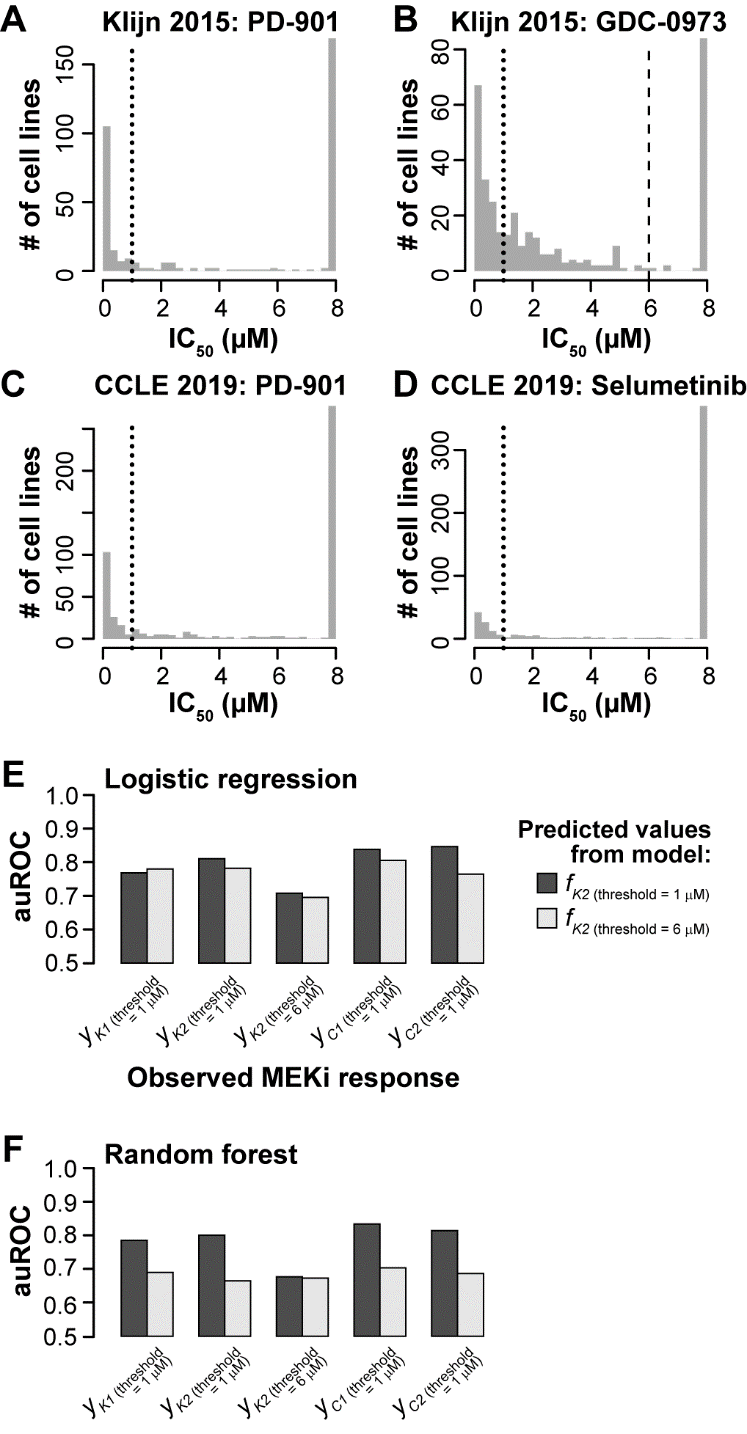


**Figure B.** Evaluating thresholds to classify cell lines as sensitive or resistant to four MEK inhibitor screens. **(A-D)** IC_50_ distributions for the four MEKi screens in Klijn 2015 (A, B) and CCLE 2019 (C, D). Vertical dotted lines: 1 μM threshold used to classify cell lines as sensitive or resistant to MEK inhibition. Vertical dashed line in **(B)**: 6 μM threshold tested for GDC-0973 in Klijn 2015. **(E,F)** Area under the receiver operating characteristic (auROC) performances calculated by comparing observed MEKi response (x-axis) with predicted MEKi response from *f_K2_* models using 1 μM (dark gray bars) or 6 μM (light gray) thresholds trained with logistic regression **(E)** or random forest **(F)** algorithms.


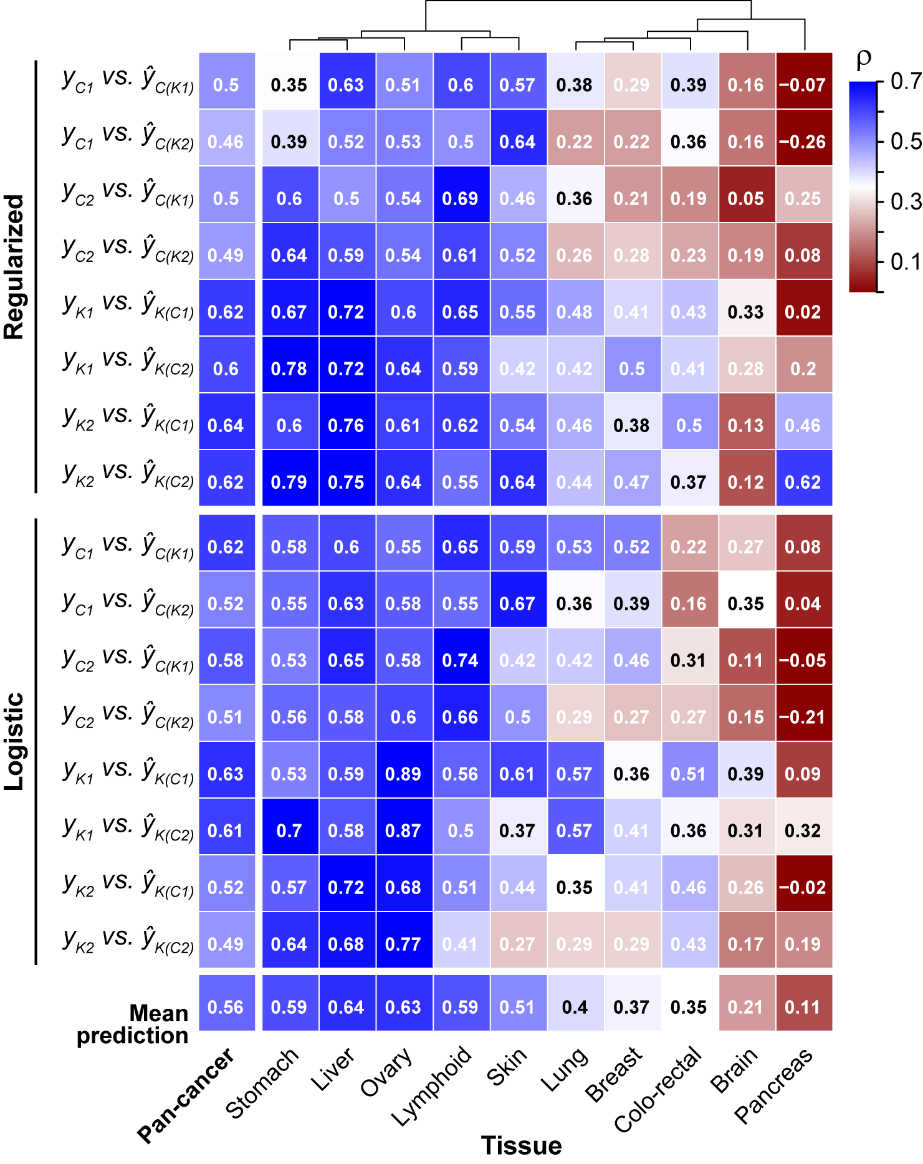


**Figure C.** Prediction performances as assessed in the full pan-cancer cell line set (left-most column) and within 10 cancer types (the next 10 columns). Heatmaps indicate rank correlation (Spearman’s ρ) between observed and predicted MEKi responses (**Figure 2**) based on regularized (top) and logistic (bottom) regression prediction models. Each row is for a specific combination of training data and test data, over two MEK inhibitors and two datasets. Also shown are the mean performance for each column (bottom row). Dendrogram at the top depicts hierarchical clustering of the tissues by their performance patterns.


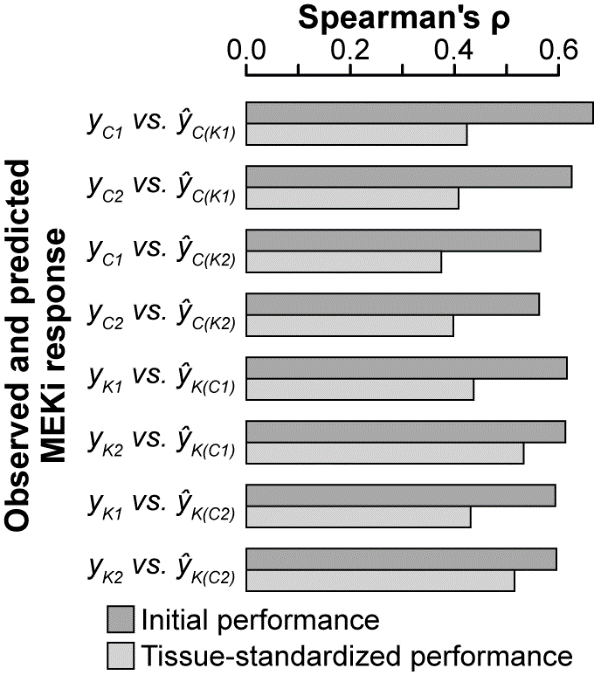


**Figure D.** Comparisons of performance of regularized regression models that included tissue-of-origin as features alongside RNA expression and DNA variant features (dark gray bars) with performances calculated following standardization of observed and predicted log(IC_50_) values within all tissues (light gray bars). Paired Mann-Whitney U test: *p* < 0.008.


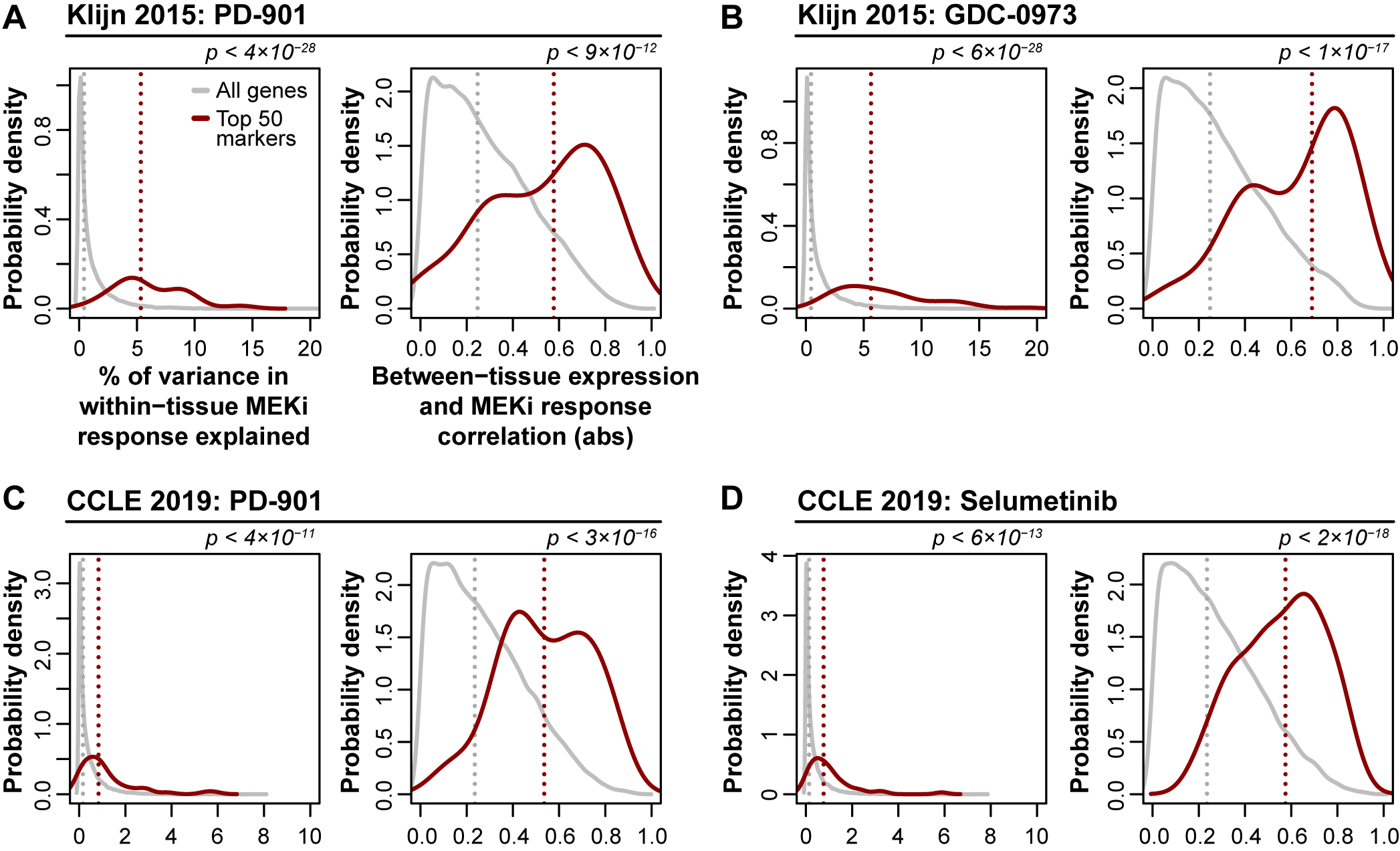


**Figure E.** Within- and between-tissue signals for the top 50 marker genes based on their maximum absolute regularized regression coefficient. (Left) Probability density distributions for a measure of within-tissue signal: the % of within-tissue variance in MEKi response explained for the top 50 markers (red) and all genes (gray). (Right) Probability density distributions for a measure of between-tissue signal: the absolute correlation (*r*) between the mean per-tissue gene expression and the mean per-tissue MEKi response. Vertical dotted lines indicate median values for color-matched distributions. *P*-values are from Mann-Whitney U tests comparing the two distributions in each panel. **(A-D)** Results are shown for the four MEKi screens.

**Table A. Additional performance metrics for regularized and logistic regression algorithms.**

| **Algorithm** | **Observed MEKi response** | **Predicted MEKi response** | **Spear-man's**  **ρ** | **Concor-dance index** | ***r^2^*** | **NRMSE^1,3^** | **NMAE^2,3^** | **AUC-ROC** |
| --- | --- | --- | --- | --- | --- | --- | --- | --- |
| Regularized | *y_K1_* | *ŷ_K(K1)_* | 0.562 | 0.747 | 0.298 | 33 | 27 | 0.778 |
| Regularized | *y_K2_* | *ŷ_K(K1)_* | 0.517 | 0.692 | 0.292 | 21.8 | 16.9 | 0.797 |
| Regularized | *y_K1_* | *ŷ_K(K2)_* | 0.511 | 0.72 | 0.263 | 33.9 | 28.7 | 0.75 |
| Regularized | *y_K2_* | *ŷ_K(K2)_* | 0.473 | 0.673 | 0.325 | 20.2 | 15.6 | 0.766 |
| Regularized | *y_C1_* | *ŷ_C(C1)_* | 0.617 | 0.784 | 0.357 | 22.2 | 15.9 | 0.85 |
| Regularized | *y_C2_* | *ŷ_C(C1)_* | 0.531 | 0.802 | 0.227 | 22.4 | 15.5 | 0.796 |
| Regularized | *y_C1_* | *ŷ_C(C2)_* | 0.621 | 0.787 | 0.374 | 21.4 | 15.5 | 0.833 |
| Regularized | *y_C2_* | *ŷ_C(C2)_* | 0.531 | 0.803 | 0.272 | 20.4 | 14.1 | 0.804 |
| Regularized | *y_K1_* | *ŷ_K(C1)_* | 0.618 | 0.77 | 0.42 | 29.2 | 24 | 0.843 |
| Regularized | *y_K2_* | *ŷ_K(C1)_* | 0.6 | 0.725 | 0.472 | 17.7 | 13.7 | 0.837 |
| Regularized | *y_K1_* | *ŷ_K(C2)_* | 0.639 | 0.78 | 0.452 | 28.7 | 23.6 | 0.863 |
| Regularized | *y_K2_* | *ŷ_K(C2)_* | 0.618 | 0.734 | 0.492 | 17.1 | 13.2 | 0.855 |
| Regularized | *y_C1_* | *ŷ_C(K1)_* | 0.501 | 0.725 | 0.315 | 22.9 | 17.8 | 0.809 |
| Regularized | *y_C2_* | *ŷ_C(K1)_* | 0.456 | 0.76 | 0.221 | 19.2 | 14.2 | 0.802 |
| Regularized | *y_C1_* | *ŷ_C(K2)_* | 0.499 | 0.725 | 0.338 | 22.2 | 17.2 | 0.816 |
| Regularized | *y_C2_* | *ŷ_C(K2)_* | 0.485 | 0.778 | 0.267 | 17.2 | 11.8 | 0.822 |
| Logistic | *y_K1_* | *ŷ_K(K1)_* | 0.65 | 0.793 | 0.391 | 31.1 | 24.8 | 0.812 |
| Logistic | *y_K2_* | *ŷ_K(K1)_* | 0.607 | 0.723 | 0.328 | 24.8 | 19.9 | 0.846 |
| Logistic | *y_K1_* | *ŷ_K(K2)_* | 0.552 | 0.742 | 0.297 | 33.5 | 27.4 | 0.768 |
| Logistic | *y_K2_* | *ŷ_K(K2)_* | 0.53 | 0.691 | 0.265 | 24.9 | 20 | 0.81 |
| Logistic | *y_C1_* | *ŷ_C(C1)_* | 0.603 | 0.778 | 0.35 | 24.8 | 18.4 | 0.844 |
| Logistic | *y_C2_* | *ŷ_C(C1)_* | 0.518 | 0.795 | 0.206 | 26.8 | 20.9 | 0.788 |
| Logistic | *y_C1_* | *ŷ_C(C2)_* | 0.48 | 0.717 | 0.261 | 26.3 | 19.7 | 0.766 |
| Logistic | *y_C2_* | *ŷ_C(C2)_* | 0.431 | 0.745 | 0.178 | 26.5 | 20.1 | 0.735 |
| Logistic | *y_K1_* | *ŷ_K(C1)_* | 0.634 | 0.774 | 0.446 | 28.3 | 21.9 | 0.848 |
| Logistic | *y_K2_* | *ŷ_K(C1)_* | 0.614 | 0.728 | 0.421 | 21.8 | 17 | 0.847 |
| Logistic | *y_K1_* | *ŷ_K(C2)_* | 0.519 | 0.72 | 0.344 | 30.8 | 24.4 | 0.798 |
| Logistic | *y_K2_* | *ŷ_K(C2)_* | 0.494 | 0.68 | 0.372 | 21.5 | 16.9 | 0.787 |
| Logistic | *y_C1_* | *ŷ_C(K1)_* | 0.618 | 0.78 | 0.343 | 24.8 | 19.8 | 0.856 |
| Logistic | *y_C2_* | *ŷ_C(K1)_* | 0.522 | 0.796 | 0.2 | 25.2 | 20.3 | 0.826 |
| Logistic | *y_C1_* | *ŷ_C(K2)_* | 0.584 | 0.763 | 0.332 | 23.6 | 18.8 | 0.838 |
| Logistic | *y_C2_* | *ŷ_C(K2)_* | 0.51 | 0.789 | 0.22 | 22.1 | 17.4 | 0.846 |

^1^ Normalized root mean squared error.

^2^ Normalized mean absolute error.

^3^ Errors were normalized to the range of observed (obs) values: *error / (max(obs) – min(obs)) * 100*, and calculated after observed and predicted values were standardized by scaling linearly between 0 and 1 and subtracting the scaled mean.

**Table B. Overlap among the top 50 regularized regression biomarkers from models *f_K1_*, *f_K2_*, *f_K3_*, and *f_K4_*.**

| **Models** | **Number of top 50 biomarkers in common** |
| --- | --- |
| ***f_K1_***, ***f_K2_*** | 15 |
| ***f_K1_***, ***f_C1_*** | 2 |
| ***f_K1_***, ***f_C2_*** | 1 |
| ***f_K2_***, ***f_C1_*** | 0 |
| ***f_K2_***, ***f_C2_*** | 0 |
| ***f_C1_***, ***f_C2_*** | 24 |

**Table C. Optimal model parameters for regularized and logistic regression algorithms.**

| **Algorithm** | **Model** | **λ ^1^** | **α ^2^** |
| --- | --- | --- | --- |
| Regularized regression | *f_K1_* | 1 | 0.1 |
|  | *f_K2_* | 1 | 0.1 |
|  | *f_C1_* | 10 | 0 |
|  | *f_C2_* | 10 | 0 |
| Logistic regression | *f_K1_* | 0.05 | NA |
|  | *f_K2_* | 0.1 | NA |
|  | *f_C1_* | 0.01 | NA |
|  | *f_C2_* | 0.1 | NA |

^1^ For regularized regression, λ was used for both feature selection and β-penalization. For logistic regression, λ was used for feature selection only.

^2^ α was used to evaluate three regularization methods: ridge regression (α = 0), elastic net (0 < α < 1), and LASSO (α = 1). *NA*: not applicable, α is not a parameter for logistic regression.

**Table D. Optimal model parameters for random forest algorithms.**

| **Algorithm** | **Model** | **# of trees** | **# of features ^1^** | **Min. node size** |
| --- | --- | --- | --- | --- |
| Random forest (regression) | *f_K1_* | 500 | 4795 (10%) | 1 |
|  | *f_K2_* | 1000 | 4795 (10%) | 10 |
|  | *f_C1_* | 500 | 23978 (50%) | 10 |
|  | *f_C2_* | 500 | 4795 (10%) | 10 |
| Random forest (binary) | *f_K1_* | 750 | 11989 (25%) | 5 |
|  | *f_K2_* | 500 | 479 (1%) | 10 |
|  | *f_C1_* | 500 | 4795 (10%) | 1 |
|  | *f_C2_* | 500 | 23978 (50%) | 5 |

^1^ Percentages indicate proportion of total features.
