# Supplementary material for: Impact of between-tissue differences on pan-cancer predictions of drug sensitivity
Source: PLoS Comput Biol. 2021 Feb 25;17(2):e1008720. doi: 10.1371/journal.pcbi.1008720 (PMC7906305; doi:10.1371/journal.pcbi.1008720)
Supplement: Attachment — Submitted filename: response_to_reviewers_v3.pdf [file pcbi.1008720.s006.pdf]

Response to reviewers

Below we have reproduced the reviewers' comments, numbered them, and provided our detailed response in blue text. We submit two versions of the revised manuscript: one clean version with all changes accepted, and another with tracked changes in Word, and comments on the side to indicate which numbered comment the change is associated with. Line numbers below refer to the version with tracked changes.

Reviewer #1:

Comment 1.1. There is a good focus on the literature review within the introduction, however, I recommend the authors to rewrite the introduction section, as some sections of it, is difficult to follow, specifically connections between biological concepts.

Thank you for the suggestion. We have revised a paragraph in the Introduction to better explain the connection between concepts, while adding three new references on the topic of tissue-of-origin on pan-cancer drug response predictions.

The revised paragraph from the revised Introduction (**lines 78-96**):

Tissue-of-origin has a complex relationship with pan-cancer drug response predictions [14,15,25–27]. Some studies reported that pan-cancer models that include all available tissues may be outperformed by those that include only a well selected subset [25,26]. Others found that both drug response and molecular features (e.g. mRNA expression levels) often vary by tissue [27]. Thus, drug response can be predicted based on tissue type alone and tissue-specific molecular properties can drive the performance of a pan-cancer model without necessarily being driven by inter-tumor differences within a cancer type [14]. It is also unclear if the prediction performance could vary among cancer types, a situation that would call for tissue-specific guidelines of applying prediction models. In clinical practice, while therapeutic decisions are often made solely based on cancer type, there is often the additional need, and potential benefit, to predict variable response among tumors within a cancer type.

Comment 1.2. “The observation that cell lines respond similarly to different MEK inhibitors indicates cross-MEKi predictions are feasible, provided the two compounds are chemically similar.” That means those who have similar drug response have similar compound structure. Did you study if other way around is also valid? Meaning that if two compounds are similar then their drug responses are similar? Or with further analysis (i.e. stringing, REFINED, OmicsMapNet, and etc) that includes fingerprints for the compounds.

We now realize that mentioning chemical similarity among drugs for the same target opens many new questions regarding the physiochemical properties of compounds and their pharmaceutical activities, a topic that warrants a separate study on its own. Ultimately, we are unprepared to address questions in medicinal chemistry. The original intention of the statement was to clarify that our modeling, while focused on tissue-tissue differences, are potentially generalizable to compounds with a shared protein target or inhibition mechanism. To avoid confusion, we have removed the clause "*…provided the two compounds are chemically similar.*" in the revised manuscript (**lines 146-147**).

Comment 1.3. Authors have claimed that “Cell lines from the same primary tissue tend to be present in similar regions of the PC1-PC2 space (Figure 1E)”. It is nice to visualize in such a approach, however the conclusion is not necessarily correct, as only for one tissue we can confidently make such a conclusion not for the rest. That may be because of information loss that embedded in any dimensionality reduction technique such as PCA. To ensure that the

conclusion is correct I urge the authors to consider applying other dimensionality reduction techniques such as MDS, Isomap, t-SNE, or so many others to evaluate if they can reach such a conclusion.

The reviewer is correct that the PC1-PC2 plot (previous Figure 1E) did not allow visualization of how similar tissues are separated. We have performed an alternative dimensionality-reduction analysis, t-SNE, which projects isolated clusters for cell lines of lymphoid, breast, and skin origin (see the figure below). Other tissue types - ovary, liver, pancreas, brain, and stomach/colorectal - also appear more tightly clustered than in PCA. In contrast, the lung cancer-derived cell lines are still highly heterogeneous.

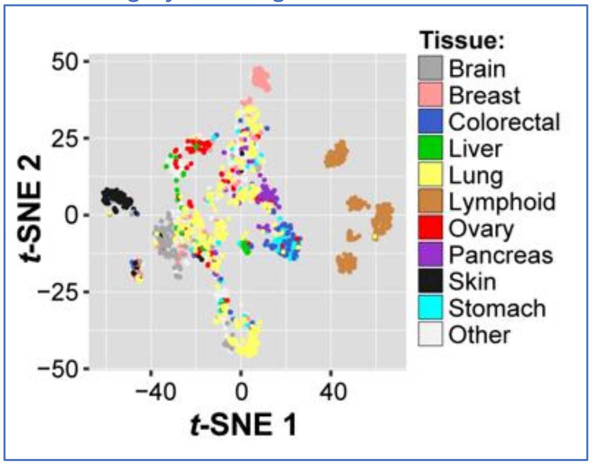

We replaced **Figure 1E** with the new t-SNE plot and moved the original PCA plots as two new panels in **Figure S1**. The associated text (**lines 148-154**) and figure legends were revised accordingly.

Comment 1.4. It was mentioned in the paper that "Cell lines from the same primary tissue tend to be present in similar regions of the PC1-PC2 space (Figure 1E) and have correlated RNA expression levels (Figure S1), highlighting that cell lines derived from the same primary tissue have similar transcriptomic features." Seems like authors are considering correlation between 0.3-0.4 as high correlation. I don't think that is a fair call, and the threshold must be larger value. On the other hand, correlation has its own problems that are well known in stat community. For instance, correlation between to signals (sin(x) and cos(x)) is zero, however we know that they are the exact same signal with some delay, which indicates correlation cannot capture some important information. For more accurate analysis, I recommend authors to consider other metrics such as Cosine similarity, R2 or mutual information, to measure similarity between RNA expressions.

Whether a correlation value in the range of 0.3-0.4 is high or low needs to be evaluated against a baseline, i.e., a null distribution. In the revised manuscript we emphasized the *comparative* nature of the analysis: cell lines from the same tissue had more similar expression patterns compared to cell lines from different tissues (**lines 154-159**). This is not a controversial statement (see for example PMID: 10963602, PMID: 12086872).

The decision of choosing a distance measure, with options such as cosine similarity, r^2 or mutual information, is also based on their relative effectiveness in a comparative setting, rather than any pre-defined thresholds. For the specific datasets we analyze, the tissue label has a major influence on the sample-sample transcriptome heterogeneity, even though different methodologies bring different appearances, as can be seen in the PCA and t-SNE plots.

Our revised sentence:
"*These results indicate that cell lines derived from the same primary tissue have more similar transcriptomic profiles than cell lines from different tissues, a pattern that is consistent with previous cancer subtype analyses [29,30]*".

Comment 1.5. Authors must explain how they pick the threshold value for dichotomizing the cell lines responses as sensitive and resistive.

Thank you for this suggestion. The IC_{50} distributions for all MEKi's examined in this study are clearly bimodal (see figure inserted below). For 3 of the 4 MEKi's, the bimodal distribution is readily split at 1 μM (indicate by the vertical line), justifying it as the threshold to define sensitive and resistant cell lines. We note that 1 μM is the threshold used in other studies for MEK inhibitors (PMID: 20215513). For the 4th MEKi screen (GDC-0973 in Klijn 2015), the IC_{50} distribution has a wider spread in the mid-range and can be split at a higher threshold, such as 6 μM. These figures were added as the **new Figure S2A-D**.

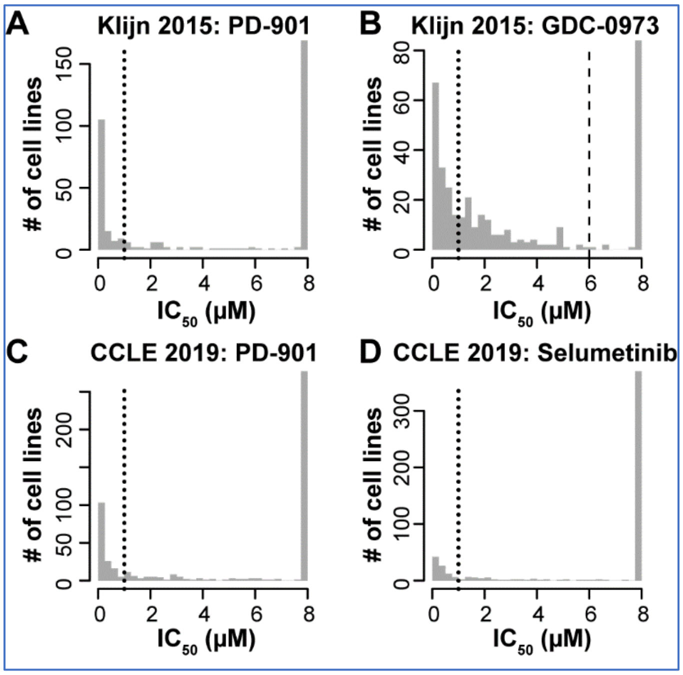

We tested if classification models (trained with logistic regression and random forest) using 6 μM as the alternative threshold could have produced different conclusions (described in the **Methods** section). We found that using the 6 μM threshold led to similar or lower performance than using the 1 μM threshold. We added these results in the **new Figure S2E-F**.

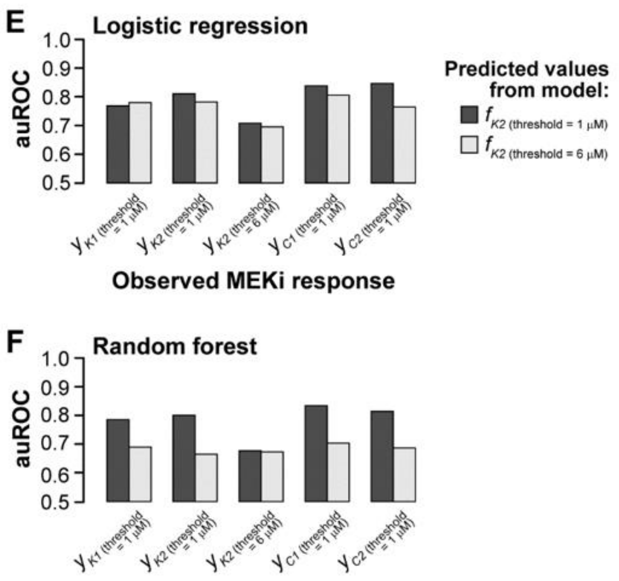

Because the original threshold of 1 performed better, we retained to the results using 1 μM in the revised manuscript.

Relevant text from the revised Methods section (**lines 557-570**):
Distributions of drug response by IC50 were bimodal for all four MEKi screens (Figure S2A-D). As a result, we tested classification-based algorithms trained with cell lines binarized as sensitive or resistant based on IC50 thresholds alongside regression-based algorithms that considered log IC50 values directly. The threshold to binarize cell lines as sensitive or resistant to a drug was set at 1 μM, a value that readily split the bimodal IC50 distributions for 3 of 4 MEKi screens and has been used previously to define cell lines as sensitive to MEK inhibition [48]. For the 4th MEKi screen, GDC-0973 in Klijn 2015, an alternative threshold, 6 μM, better separated the IC50 distribution. For the GDC-0973 screen in Klijn 2015, we generated classification-based prediction models using both 1 μM and 6 μM IC50 thresholds. We found that models trained using the 6 μM threshold performed similarly or worse than those using a threshold of 1 μM (Figure S2E,F), depending on the MEKi screen predicted. We therefore opted to use the results from the 1 μM threshold classification prediction models for GDC-0973 throughout the study.

Comment 1.6. Authors must report NRMSE, NMAE, and R2 for their predictive models, as one model can have high correlation value and high error value at the same time. On the other hand, the scatter plot of the models in figure 2C doesn't show a good performance by the models. For instance, there are so many observed values of log(IC50) = 2 that are predicted ranging from -5 to 5.

We have added a **new Supplemental Table 1** to include the requested performance metrics, NRMSE, NMAE, r, r^2 and concordance, for the regularized and logistic algorithms (described in **lines 205-208**). These new metrics led to similar results and the same conclusions as those we used, rho and auROC.

A technical note: The distribution of the observed IC_{50} values varies by drug and dataset, and the distribution of predicted values varies by algorithm and dataset, making it all the more important to focus on the comparative patterns rather than the high and low of absolute values.

For the calculation of NRMSE and NMAE, we standardized both the predicted and the observed drug response values. Standardization was performed by scaling the values linearly from their original range to the interval of [0,1], and then subtracting the scaled mean.

Comment 1.7. How do you perform auROC for a regression task? Could you please show a reference for that? As far as I know auROC is only possible when you have probability value for a classification task.

Thank you for raising the question that a regression task is rarely evaluated by auROC. While we followed the usual practice of reporting rho for regression models and auROC for classification models, we also wanted to compare between these two classes of models. For this purpose, we made the unusual decision to add the reporting of auROC for regression and rho for classification tasks.

To answer the reviewer's question: for the regression models, auROC is calculated based on the ordered series of 2-by-2 count matrices of true and false positives and true and false negatives, iterated through the range of predicted values. Using the top panel of Fig 2C as an example, each point on the ROC was calculated by setting a predicted log(IC50) as the threshold for predicting sensitive and resistant samples, to populate a 2-2 counts table and calculate the FP and FN rates.

Comment 1.8. Authors should mention which statistical test did they use for the “Between- and within-tissue performance of pan-cancer MEKi response predictions” section.

The p-values in the first paragraph were for testing if the rank correlation coefficients, rho, between the observed and predicted tissue-average IC50 values, are at 0 (null hypothesis) or non-0 (alternative hypothesis), given the number of observations (n=10 tissue types). The p values were calculated by using the *cor.test* command in R. We added this detail to the revised Results section (**line 226**).

In the third paragraph, we reported how the rho values were consistently reduced from one set of modeling results to another set, for either 8 models (Figure S4) or 16 models (Figure 4C and D combined). Here the p values were based on Mann Whitney paired U tests, as implemented in the *wilcox.test* command in R.

In the fourth paragraph, we tested if the distribution for the 50 markers are different from that of all the other markers (applied four times, for Figure S5 A-D, the left panel). We applied the non-parametric Mann Whitney U test.

Comment 1.9. In the section where authors investigated “if pan-cancer prediction models can outperform those generated by considering a single cancer type.”, there are so many avenues for improvement as it is very interesting to perform such an investigation, however authors couldn’t achieve superior performance. To this end, I recommend authors to perform some other non-linear regression model such as Random forest, XGBoost and so on. Also perform robustness analysis similar to the community effort paper: https://www.nature.com/articles/nbt.2877

Our plan is to *compare* performance for similar algorithms under similar settings, without seeking a threshold for claiming "superior performance" in the absolute sense. We explored eight model combinations to cover the avenues commonly used in predictive modeling, including random forest, with the main interest in comparing pan-cancer models with tissue-

specific models. Whether one model is "superior to" another depends on the tissue type and the four dataset combinations, as shown in Figure 5. The reviewer's suggestion, if we understood correctly, is to explore additional avenues to see if the performance, for either pan-cancer or tissue specific models, can be improved further. We agree that there are many worthy directions. However, at this point the revision already added many new results, and we do not feel it is feasible to add more analyses.

On the reviewer's excellent suggestion of assessing robustness, we have (1) previously used down-sampling to evaluate the impact of sample size, and (2) added a parameter-sweep analysis over a grid of parameter values, as explained below in response to Comment 2.2.13.

Comment 1.10. In the “Estimating sample sizes required for optimal prediction performance” section, an interesting investigation is provided, however authors can improve the estimation power on the sample size by performing evaluation on increasing the sample size in addition to reducing it. For instance, one can simply perform boot strap sampling or more advanced data augmentation methods: https://link.springer.com/article/10.1007/s12065-019-00283-w

We performed the suggested analysis by up-sampling of instances (i.e. sampling with replacement), with $n_{instances}$ = 300, 500, 750, and 1000. We trained models using one MEKi response series from the CCLE dataset (y_{C1}) along with the CCLE features (x_C), applied the models to the Klijn 2015 dataset (x_K), and evaluated performance by comparing with the Klijn 2015 PD-901 MEKi response (y_{K1}). The Spearman’s rho values for n=300 was 0.58, which did not increase with larger samples: rho=0.49, 0.56, and 0.58 for n=500, 750, and 1000, respectively. This result, that bootstrap up-sampling did not increase performance, is not surprising as the approach merely replicated some of the samples to increase apparent sample size, without increase the number of unique instances. We do not discuss this analysis in the revised manuscript.

__**Reviewer #2:**__

This is a quite interesting and well-written manuscript, that was a pleasure to read, including a number of useful conclusions and lessons for the researchers who are developing drug response prediction models in cancer cell line panels. This reviewer especially enjoyed the last results section and conclusions at the end of discussion about the required number of cell lines to construct a robust drug response prediction model. However, there remain several issues that will need to be addressed (see below) to make this work more rigid in terms of the statistical analyses, and even more useful for the computational biology community.

Major comments:

Comment 2.1.1. Although the results are interesting, these are not entirely novel. The authors should put their results and conclusion into the context of previous related works (e.g., PMID: 27444372, PMID: 26274927, PMID: 29016819, PMID: 29186355, PMID: 31208429, PMID: 30704458). They should also reword lines 77-80 in the Introduction and cite the existing studies on this topic. In the discussion, please state whether your results confirm previous observations for tissue-specific contribution to pan-cancer modelling, and what are the novel findings from this work, compared to the previous investigations.

We thank the reviewer for providing a particularly useful list of references, all of which have been added to a revised Introduction section, as refs 14, 15, and 24-27. PM31208429 and PM30704458 have also been added to the revised Discussion.

The new paragraph in Introduction (**lines 78-96**) is copied here:

Tissue-of-origin has a complex relationship with pan-cancer drug response predictions [14,15,25–27]. Some studies reported that pan-cancer models that include all available tissues may be outperformed by those that include only a well selected subset [25,26]. Others found that both drug response and molecular features (e.g. mRNA expression levels) often vary by tissue [27]. Thus, drug response can be predicted based on tissue type alone and tissue-specific molecular properties can drive the performance of a pan-cancer model without necessarily being driven by inter-tumor differences within a cancer type [14]. It is also unclear if the prediction performance could vary among cancer types, a situation that would call for tissue-specific guidelines of applying prediction models. In clinical practice, while therapeutic decisions are often made solely based on cancer type, there is often the additional need, and potential benefit, to predict variable response among tumors within a cancer type.

Below we explain in more detail how we assessed each reference for its relationship with our study and clarified the novelty of our study in the context of each.

- **PMID 31208429** has the greatest conceptual similarity with our study. The authors evaluated a range of model-building and feature-selection techniques for pan-cancer models of cytotoxic drug response. Importantly, they found that tissue-of-origin label is a major factor in pan-cancer prediction models, a conclusion similar to what we arrived at independently (without being aware of this study). Nonetheless, the novelty of our study are 1) providing a framework to estimate the effect size of between-tissue signals on pan-cancer analysis (e.g. the Cigar Plot), 2) documenting how pan-cancer prediction performance varies across cancer types – an observation of practical value in the clinic, 3) studying the between-tissue effects for a targeted therapy (MEKi), whereas PM31208429 was on generalized cytotoxic therapies, 4) comparing pan-cancer prediction models with tissue-specific models, and 5) estimating the number of cell lines required to saturate prediction performance.
- **PMID 27444372** is a review of cancer drug response prediction models. It mentioned many of the same considerations regarding tissue-of-origin effects as we addressed, such as asking whether drug responses should be formulated in a tissue-specific or pan-cancer manner. However, it did not specifically answer these questions.
- **PMID 26274927 and PMID 29186355** discussed the effects of tissue type on pan-cancer prediction models but focus on how tissue-of-origin effects can reduce the performance of pan-cancer predictions when pan-cancer datasets are not properly stratified. **PMID 26274927** describes an algorithm to separate biomarkers that are cancer type-specific from those that are shared across cancer types, while **PMID 29186355** discussed how the expression of a biomarker (ERBB2 protein) correlates with sensitivity to lapatinib in a subset of cancer types (breast and ovary), but is not a useful predictor across all cancer types. Thus, both studies suggested that identifying the appropriate multi-cancer subsets can be a critical consideration. By contrast, our study focuses on tissue identity as the main feature, rather than considering a specific molecular feature stratified by tissue identity.
- **PMID 29016819** described how most drugs show tissue-specific sensitivity. In our manuscript, we describe how the tissue effects in pan-cancer models will contribute to prediction performance for drugs with sensitivity that varies by tissue. Therefore, PM29016819 is an important reference to cite, one that suggested that these effects may contribute to most pan-cancer drug response predictions, regardless of the drug.
- **PMID 30704458** described an interesting approach to drug response predictions using the large sample size provided by the TCGA dataset to develop deep learning autoencoders that are then used in pan-cancer drug response predictions. This study did not address how tissue-of-origin effects may contribute to pan-cancer prediction performance.

Comment 2.1.2. Many of the results are based on Spearman rank-correlation, which is a robust measure of rank association between observed and predicted responses. However, since the significance of correlation effects sizes depends on the number of cell lines, correlation values are not directly comparable when comparing pan-cancer and tissue-specific models based on different cell line numbers. It is therefore important to always specify the p-value for the correlations. One can also plot -log (p) to make the comparisons easier to interpret statistically in those plots where the sample sizes are different.

The reviewer is correct that the ***significance** of correlation effects sizes depends on the number of cell lines*. A lower sample size tends to have larger estimation errors of the correlation effect size, but does not necessarily bias towards larger effect sizes per se. In this manuscript, even though pan-cancer models were trained on a larger number of cell lines than tissue-specific models, the analyses have taken sample size into account. As described above in the response to Comment 1.8, there are at least two scenarios:
1. When assessing if a correlation value is significantly above 0, the number of samples used in calculating the correlation is incorporated into the test.
2. When assessing if one set of models out-performances another set of models, such as when comparing tissue-specific and pan-cancer models, the correlations were calculated using the same sample size, i.e, the number of cell lines for each tissue type (e.g. **Figure 5**). Here one group of rho's are directly comparable to the second group of rho's. The reviewer is correct to express a concern whether such a comparison was based on different sample sizes.

Comment 2.1.3. The authors need to better justify why AUC (not auROC) and rank correlation were used for binary and continuous prediction problems, respectively. Concordance index (CI) would provide an alternative evaluation metric that can be used in both setups, and would make the comparisons easier to interpret (e.g., Fig. 4C,D). The use of rank correlation in Figure 3 is bit misleading (the same for Figure 4A,B), since Spearman correlation considers variation of ranks, not absolute values or linear fits, like illustrated in these figures. The authors should use Pearson correlation or coefficient of determination in these plots.

This question was partially addressed in our response to Comment 1.6. We have calculated additional performance comparison metrics for the regularized and logistic algorithms, including concordance, r, r^2, normalized root mean squared error, and normalized mean absolute error. These metrics are provided in the new **Table S1**. For Figure 4A-B we initially chose ρ as it is more robust to outliers. We agree with the reviewer that since the fitted lines are from linear modeling, not robust linear modeling, it is better to use r rather than ρ. We have replaced ρ with Pearson's r in cases where lines of best fit were plotted (**Figure 3** and **Figure 4A-B**). The r values are similar to the ρ values.

Comment 2.1.4. The section "Sample size advantage…" gives a more direct comparison of pan-cancer and tissue-specific models, where the latter are trained using cell lines from each tissue only. Fig. 5 is a nice comparison, but the authors should specify the number of cell lines used for each tissue-specific models on its x-axis. Also, instead of marking p_1/2, please align p-values to the two columns being compared. The authors should also make one main figure for the comparison of the model features. Suppl. Fig. 3 style is bit hard to access, and simple Venn diagrams might work better for showing overlapping features.

We have edited Fig 5 as requested. First, the number of cell lines used to train tissue-specific and downsampled pan-cancer prediction models are now included on the x-axis in Panel A. Second, we added bars indicating the two groups being compared and generated the p-values.

We agree with the reviewer that the comparison of the top 50 model features with the other features, previously in Figure S3, warrant inclusion as a main text figure. We have added this comparison for PD-901, in boxplots, as **new Figure 4 F** and **H** (shown below). Figure 4F and 4H corresponds to the left and right panels, respectively, of Figure S3-A (the **new Figure S5-A**). The **new Figure 4G** is for showing example calculations for **Figure 4H** for three features. We revised the paragraph discussing the analysis to provide greater detail (**lines 265-283**).

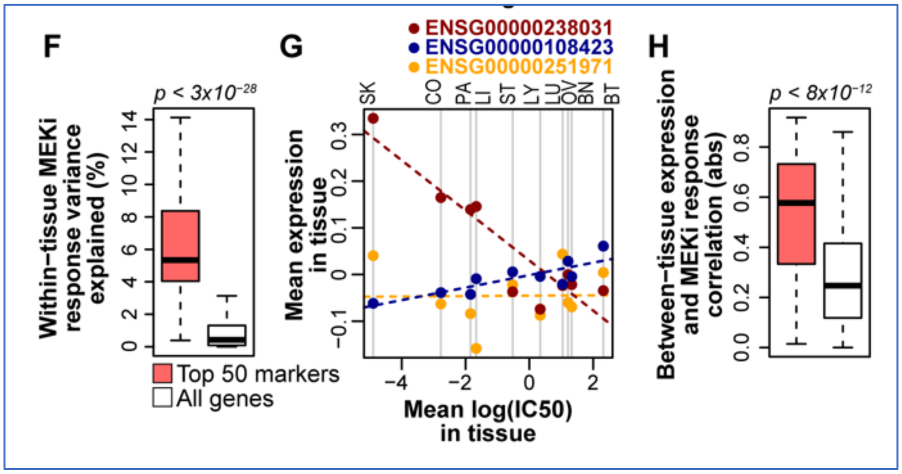

We also generated a **new Supplemental Table 2** displaying the overlap in top 50 biomarkers among the four regularized regression models (**lines 281-283**).

Comment 2.1.5. The current results focus only on three MEK inhibitors (PD-0325901, GDC-0973 and Selumetinib), out of which only two are shared between the two datasets. The authors are recommended to extend these analyses also other classes of inhibitors to guarantee that the conclusions they make in the end are generalizable also to other drug and target classes. If needed, there are also other large-scale drug testing datasets available, e.g., DGSC and CTRP v.2, which include many dugs and various molecular profiles. The authors could check from PharmacoDB suitable datasets for other kinase inhibitors.

We agree that this study can be expanded with additional drugs and datasets, similar to Comment 1.9, suggesting that we test additional models/algorithms. To keep the scope manageable, we decided to only take up one of suggestions: to examine the generalizability of our results to other drugs. We added a new analysis to quantify the proportion of variance of drug response accounted for by tissue types for the 29 drug screens in the Klijn 2015 and CCLE 2019 datasets. As shown in the newly added **Figure 4E**, the 4 MEKi screens ranked 3, 4, 6, and 8 among the 29 drug screens, significantly enriched in the high end ($p < 0.02$, U test). Thus, we expect that the drugs with comparable rank (such as those ranked among the top 10) as MEKi screens would have a similar impact of between-tissue effects on pan-cancer response predictions. We have added this analysis in the Results section (**lines 261-264**).

Specific comments:

Comment 2.2.1. Figure 1C. Statistical testing of the number of sensitive cell lines would make the plot more convincing. Please also justify why threshold of IC50 = 1uM was chosen to determine if a cell line is sensitive to a drug. Ideally, such threshold should depend on a specific drug, relative to other drugs in the cell line.

Regarding statistical testing of the number of sensitive cell lines in **Figure 1C**, the null hypothesis is that the frequency of sensitive cell lines are the same across the 10 tissue types. For each of the 4 drugs we used the 2 x 10 contingency table of cell line counts to conduct a χ^2 test of independence. We found that the frequency of sensitive lines varied significantly between tissues ($p < 2x10^{-9}$ for all 4 drugs). The statistical tests are added to the revised Results (**lines 132-133**) and to **Figure 1C**.

Regarding the justification for the 1 μM threshold for IC50, please see the response to **Comment 1.5**.

Comment 2.2.2. Figure 2D. Comparison of predictions across datasets (red and pink symbols) and within datasets is borderline significant. It would be good to analyze this bit further, as it would be quite surprising result if these prediction accuracies are overall similar, as is currently stated in the results (lines 185-187).

In the initial submission, the statistical tests were separated by dataset, so that within- and between-dataset performances in the Klijn 2015 dataset were tested separately from those in the CCLE dataset. We updated our comparison so that within- and between-dataset performances for both datasets were tested together. Based on this updated analysis, we find a significantly higher performance by Spearman's rho ($p < 0.04$). We have updated the discussion in the main text to reflect the new analysis (**lines 210-213**).

Comment 2.2.3. Figure 3. The "cigar plots" are very illustrative but they only show the mean observed and predicted log(IC50) from around 0 to 1. However, according to Figure 2C, the observed log(IC50) can be up to 2 and the predicted log(IC50) can be up to 5. Why is there so much difference between the two plots?

The mean observed log(IC50) values for the 10 tissue types, and similarly the mean predicted values, were scaled linearly from their original range to the range between 0 and 1 prior to plotting the cigar plots. This was a global transformation for all cell lines from the 10 tissues, for the x values and separately for the y values. The purpose of the scaling is to prevent the ellipses from being distorted because the observed and the predicted values are not in the same range. We have updated the figure to include "(scaled)" in the axis labels.

Comment 2.2.4. Figure 6. This is a nice analysis, but may give somewhat simplified view of the pan-cancer model performance as its prediction performance may not only be related to the sample size, but also to the correlations between the tissue groups. This should be further investigated, or at least discussed.

Thank you for this insightful comment. The performance of the pan-cancer model does indeed depend on the composition of the tissue types, the number of cell lines in them, and the "distance" among tissue types for both their drug sensitivity and their molecular features. The quantitative results we reported are therefore influenced by these characteristics of the CCLE and Klijn datasets. We have revised the Discussion to include the following: "*However, more or fewer cell lines will likely be required to saturate prediction performances based on the number of cell lines present for each tissue, as well as the relationships between the tissue groups.*" (**lines 366-368**).

Comment 2.2.5. Even if the language is good, there are certain bit cryptic sentences and terms that needs to be made more specific; for instance "group" on lines 92 ("contributions of both

group and individual identity”), and line 215 (“this is because the two tissues, at the group level”); please reword and make clearer.

In this study "tissue", "tissue type" and "group" are synonymous to each other. For the two examples highlighted by the reviewer: 1) we have clarified that group indicates tissue (**line 104**), and 2) we have removed group as the concept as it is not necessary for the point we are making (**line 244**).

Comment 2.2.6. Abstract and page 12: “a 22% decrease” is difficult to be understood. What does a 22% decrease in the Spearman correlation coefficient mean? Before and after correlation would be better. Further, since this is an average number, confidence interval or a range of correlations should be reported, too.

We agree that % decrease in a correlation coefficient can be difficult to interpret. As suggested, we have replaced the % decrease language with before-and-after range of performance in the Abstract (**lines 43-44**) and Results (**lines 252-253**).

Revised Abstract text: *Between-tissue differences make substantial contributions to the performance of pan-cancer MEKi response predictions, as exclusion of between-tissue signals leads to a decrease in Spearman’s ρ from a range of 0.43-0.62 to 0.30-0.51.*

Revised Results text: *Compared to the initial performance, the rank correlation coefficients using tissue-standardized observed and predicted log(IC50) values were reduced from a range of 0.43-0.62 to 0.30-0.51 (paired U test, $p < 0.008$), depending on the algorithm and training/testing data (**Figure 4C, D**).*

Comment 2.2.7. Abstract “RNA, SNP and CNV data”; these needs to be made more explicit when first time used, e.g., “mRNA expression, point mutations and copy number variation”; the authors should mention in discussion that also other data (e.g. methylation and proteomics) are being used in prediction models.

We have updated the wording in the Abstract to reflect the reviewer’s suggestion (**lines 38-39**): *Here, we built a series of pan-cancer models using two datasets containing 346 and 504 cell lines, each with MEK inhibitor (MEKi) response and mRNA expression, point mutation, and copy number variation data […]*

We have added in Discussion the point that additional data types, while not present in our study, may be added when available (**lines 348-351**): *Pan-cancer drug response predictions can also incorporate additional data types, such as methylation and proteomics data. We expect that prediction performances based on any data types that vary with tissue-of-origin will be similarly affected by both between- and within-tissue signals.*

Comment 2.2.8. Lines 59-60: For within a cancer type prediction model, the authors should cite such prediction models, e.g., the DREAM7 drug sensitivity prediction challenge in breast cancer cell lines (PMID: 24880487), which is still consider a state-of-the-art in the field of drug response prediction in cancer cell lines.

We have added the DREAM7 reference to the introduction and to the section “Sample size advantage of pan-cancer models over tissue-specific models” in Results (Reference #13 in the revised manuscript).

Comment 2.2.9. Page 12: the first sentence "five tissues whose within-tissue variability was accurately predicted". Please define what does it mean to be “accurately predicted“? For instance, specify a threshold for the Spearman correlation coefficient to determine whether the prediction is accurate or not.

We have added a stated threshold of mean rho > 0.5 for this analysis (**lines 232-234**), which was based on the clustering of the within-tissue prediction performance in **Figure S3**.

Comment 2.2.10. Line 119: Between- and within-tissue performance of pan-cancer MEKi response predictions section. Please emphasize whether these results are based on the pan-cancer model or tissue-specific model (c.f. the next section: Sample size advantage…)? It seems the former, but good to specify in the text.

The reviewer is correct that the results are from the pan-cancer predictions. As suggested, we have clarified that the results are from pan-cancer models in **lines 223 and 229**.

Comment 2.2.11. Line 358: ”We further standardized RNA expression data for each gene by linearly scaling values across cell lines to a range between 0 and 1 and shifting the scaled values by subtracting the scaled mean”; the rationale of this post-processing remains unclear and needs to be justified in the Methods section.

We standardized RNA expression levels for each gene, so that genes are present in similar scales and can contribute comparably to prediction models. In addition, standardization brings the RNA data from Klijn 2015 and CCLE 2019 datasets into similar ranges, allowing a model from one dataset to be effectively applied to the second dataset. We have added this detail to Methods (**lines 432-435**).

Comment 2.2.12. Methods. Please give more details of the assays of the two studies. For instance, SNP arrays and exome-seq are quite different for detecting point mutations. How does that affect the results? Were the same cut-offs for gene amplifications and deletions used in both studies. No details of the drug assays provided.

We have added additional details as suggested.

For the drug assays, we included information on replicates and drug dose ranges, and discussion of the challenges of using IC_{50} as a drug response metric (**lines 410-419**):
Klijn 2015 tumor cell lines were screened with 3-4 replicates across 9 drug doses (range: 0.15 nM to 20 μM); while the CCLE cell lines were screened with at least 2 replicates across 8 drug doses (range: 2.5 nM to 8 μM). The resulting dose response curves were summarized with the IC_{50} value: the dose at which 50% of cells are non-viable; lower IC_{50} values indicate greater sensitivity. IC_{50} can be difficult to interpret, as it does not account for different shapes in dose response curves or for differences in minimum viable cells observed. However, IC_{50} was used in this study as it was the sole drug response measure in common between the Klijn 2015 and CCLE 2019 datasets.

We added more details for the DNA variants, noting that the difference in the number of SNP calls is tempered in part due to aggregating SNP and CNV data to the gene level (**lines 474-478**):
The CCLE dataset contains more than 5 times as many missense and nonsense SNP calls than the Klijn 2015 dataset, which is largely due to the different platforms employed for genotyping

(i.e. SNP array vs. exome-seq). The effects on downstream analysis brought by differences in the total number of SNP calls between the two datasets is tempered by mapping of SNPs to the gene level.

Last, we added the threshold used in the CCLE dataset for calling gene amplifications and deletions from SNP array data (Klijn 2015: **line 465**, included in initial submission; CCLE 2019: **lines 483-484**). The thresholds for amplifications and deletions were 1 and -0.75, respectively, for Klijn 2015, and 0.7 and -0.7, respectively, for CCLE 2019.

Comment 2.2.13. In the binary problem, the authors use logistic regression with LASSO to first select features. Instead of that, logistic LASSO regression might be a better and more straightforward option which is also implemented in the glmnet package. The authors should consider using that in the revised work.

We followed reviewer's suggestion to apply a regularized logistic regression approach using the glmnet package, selecting optimal alpha and lambda values with a parameter sweep within the training set (alpha options: 0, 0.25, 0.5, 0.75, and 1; lambda options: 0.001, 0.01, 0.1, and 1; 4x5=20 total parameter sets tested). When the logistic regression models were applied to validation sets and across datasets, we found a near-identical performance compared with the original logistic regression with LASSO feature selection approach (see plot). Because the performance is similar, we opted to retain the logistic regression results currently in the text (i.e. not replaced with regularized logistic regression results).

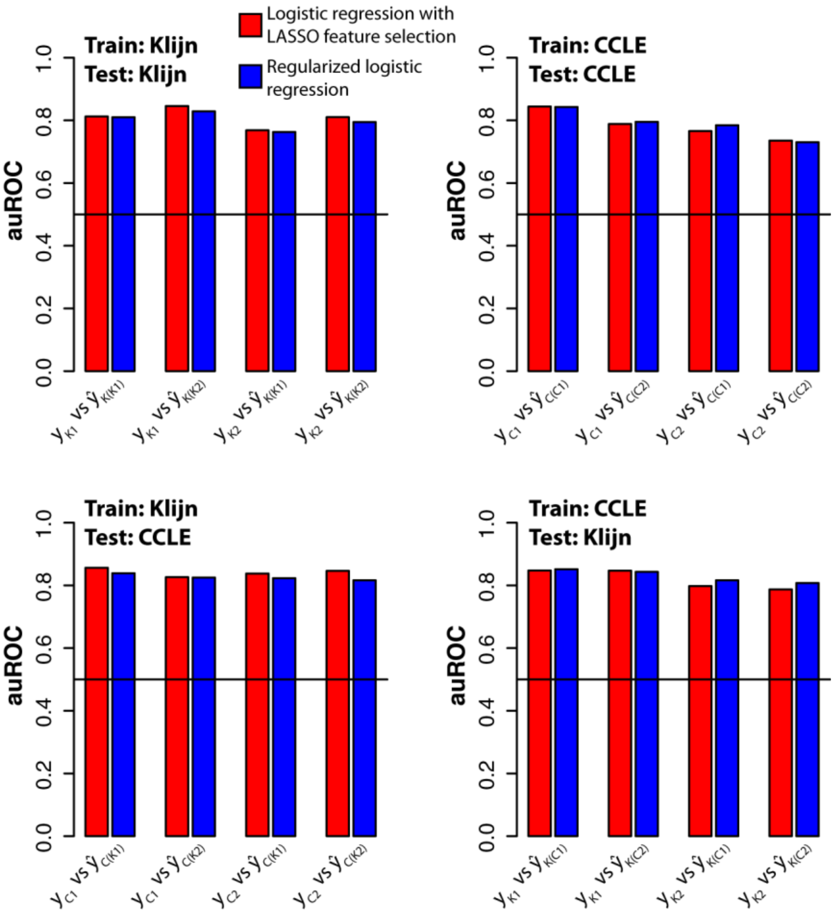

Comment 2.2.14. It has been shown that cancer tissue type may directly contribute to the prediction of drug sensitivity in the pan-cancer models. It remained unclear whether the tissue-of-origin was used as predictor in any of the analyses; and if not, the authors need to explain why it was not included in any of the models?

We generated a new set of models using regularized regression that, along with the RNA expression and DNA variant features, added the 11 binary features for tissue-of-origin. These models performed similarly to those without the tissue-label features (p = 0.74, paired Mann Whitney U test). As before, performance is reduced when the observed and predicted drug

response values were standardized within tissue ($p < 0.008$, paired U test). That inclusion of tissue label does not lead to increased performance is not surprising, as tissue type is aligned with a subset of molecular features, thus adding tissue label did not add much new information. We described this new analysis in Results (**lines 255-261**) and added a new **Figure S4**.

Comment 2.2.15. Supplementary Table 1 shows boundary lambda in model f_C1 and model f_C2 for the regularised regressions. Please re-tune these parameters. Further, it remains unclear whether the parameters of random forest regression were optimized or not, including "number of tree to grow", "cutoff", etc?

We ran an additional parameter sweep for the f_{C1} and f_{C2} regularized regression models, testing lambdas of 1, 10 (original max), 100, 1000, and 10000 with alphas of 0 (original max), 0.1, and 0.2. Thirty models for parameter sweeps were generated for each drug, each considering a randomly-selected 70% of cell lines as the training set. For each of the thirty models, the 30% of cell lines not selected for model-building are used to assess performance, using Spearman's rho, and the mean rho of the 30 replicates are used to compare across parameter sets. Based on this analysis, for the f_{C2} model, lambda = 10 and alpha = 0 remained as the optimal parameter set. For the f_{C1} model, an alternative parameter set, lambda = 1, alpha = 0, had optimal performance. This is somewhat surprising, as this parameter set was available in the initial sweep. We think there are likely multiple sets of parameters that provide similar, near-optimal performance, and that the final optimal set is influenced by the random selection of training instances during the parameter sweep model building. As a result, we have opted to maintain the original parameter set of alpha = 0 and lambda = 10 for the f_{C1} model and have added the discussion of this analysis with additional lambda parameters in Methods (**lines 592-602**).

In addition, we re-ran the random forest analysis by tuning 3 additional parameters: 1) number of trees, 2) number of features available as candidates at each tree split, and 3) node size, which affects tree size/depth. With the updated random forest models, we found similar results as before, where random forest performs similarly to regularized and logistic regression, except for f_{C2}, which has lower performance than f_{K1}, f_{K2}, and f_{C1}. We have updated **Figure 2D** to reflect RF performance with additional parameter tuning, updated the Methods accordingly (**lines 575-590**), and added a new **Table S2** to list the optimal parameters.

Reviewer #3:
Inter-tumor heterogeneity in molecular characteristics and phenotypes are well known, but analyzing pan-cancer dataset may yield a uniformly predictive molecular signature among different cancers for certain cancer drugs. Lloyd et al. studied the impact of inter-cancer heterogeneity and intra-cancer heterogeneity on pan-cancer predictions of drug sensitivity. Authors extensively explored the performance of the pan-cancer predictions only for one targeted cancer drug family, MEK Inhibitors. **[Comment 3.0] I thus strongly suggest authors to publicly share their analysis codes and datasets used in this study that will enable oncologists and cancer biologists to explore inter-cancer heterogeneity in responses to other anti-cancer therapeutics. [/Comment 3.0]** in the manuscript, there are paragraphs that need more description and clarification. Please find my comments below.

We agree completely that the community benefits from access to the codes and the processed datasets. Toward this goal, we have made available the codes needed to run the model-building and parameter sweeps through GitHub (*https://github.com/johnplloyd/R_prediction_model_building*).

Additionally, processed drug responses and molecular features (mRNA expression and DNA variants) used in our analyses have been uploaded to the CyVerse Data Store (*https://de.cyverse.org/dl/d/43AB0125-4826-4599-9337-E8B61F41DBA4/lloyd_etal_pancancer_MEKi_processed_data.zip*).

Links to these resources are provided in (**lines 491-494 and 590-591**) of Methods.

Comment 3.1. Page 6: Line 126-129: Figure 1D. For Drug response correlation, a correlation coefficient of PD-901 sensitivities between two cell line panels of 0.81 is lower than correlation coefficients of two different drugs in each of the two studies (r=0.88 between PD-901 and GDC-0973 in Kijin and r=0.83 between PD-901 and Selumetinib). Does the lower correlation coefficient for the same drug imply a larger study-specific batch effect on drug sensitivity measurements?

There are two possible explanations for the lower cross-dataset correlation of PD-901 than the within-dataset correlation between PD-901 and GDC-0973. First, as suggested by the reviewer, there could be study-specific technical differences in measuring IC50, including differences in cell viability assays, and the dose and duration of the drug exposure. Second, cell lines continue to evolve when they are kept in different laboratories, and their drug sensitivities may have changed over time, producing genuine biological differences for the same cell lines measured in different laboratories. We have updated Results to discuss these possibilities (**lines 141-145**).

Comment 3.2. Page 7: Line 144-146: Please describe a rationale for a thrould of IC50 <=1nM with which cell lines were stratified as sensitive to a drug.

Please see the response to **Comment 1.5**.

Comment 3.3. Page 7: Line 148: Is logistic regression analysis a regularized logistic regression?

No, the logistic regression itself was not regularized, but it was preceded by an upstream feature-selection step using LASSO. As discussed in response to **Comment 2.2.13**, we ran a regularized logistic regression, and found similar performances compared to logistic regression with LASSO feature selection (see plot in 2.2.13).

Comment 3.4. Page 10: Line 187-189: In Figure 1D, there are many triangles (cross-MEKis prediction) with lower correlation coefficients and auROC values than circles (same MEKi). Although p-value is above 0.05, I don't think it is reasonable to argue that cross-MEKis performed comparably.

In the initial submission, the statistical tests were separated by dataset, so that the Klijn 2015 performances were tested separately from those in the CCLE dataset. We updated our comparison so that within- and cross-MEKi performances for both datasets were tested together. Based on this updated analysis, we find a marginally-significant higher performance for within-MEKi comparisons by Spearman's rho ($p = 0.10$). We have updated the Results to reflect this analysis (**lines 213-217**).

Comment 3.5. Page 10: 187-190: please specify the ranges of observed spearman correlation coefficients and AUCs in addition to the reported U-test p-values.

We have added the means and ranges associated with the U tests in the revised text (**lines 215 and 216**).

Comment 3.6. Page 12: Line 204-206: I wonder how many cancer cell lines were originated from those five cancer types in the pan-cancer training dataset. These five cancers may account for the majority of cell lines in the training dataset and show a good prediction performance.

These five cancer types account for roughly half of the cell lines in the training datasets (see **Fig 1B**). As a result, only a portion of the overall performance can be attributed to the accurate within-tissue signals for these five tissues.

Comment 3.7. Page 12: Line 213-216: Please describe How you choose these two cancer types? In Figures 4A and 4B, breast cancer cell lines had truncated IC50 at around 2. It may not make sense to calculate correlation coefficients with this truncated drug sensitivity data.

For **Figure 4A-B** the goal was to illustrate how between-tissue predictions can impact overall prediction performance. To create an extreme example, we selected a pair of tissues, with one generally sensitive and the other generally resistant, but neither tissue had successful within-tissue predictions. Increased prediction performance across the two tissues (e.g. black dotted line in Fig 4A) would therefore be mainly due to the between-group differences of the two selected tissues. In other words, a prediction considering these two tissues together provides little additional information than knowing the tissue-of-origin.

Comment 3.8. Page 12: Line 215-217: To argue this, i think authors need to show that combining two nearest cancer types on PC values and IC50 such as colorectal and stomach does not improve an overall prediction performance.

As explained in the response above, our goal is not to provide a generalized example, rather an extreme example to illustrate how tissue characteristics may contribute to overall prediction performance in a pan-cancer setting. We have revised the wording in this section to clarify that the two-cancer type comparison of brain and pancreas was driven almost solely by between-tissue signals (**lines 245-246**). We followed up this extreme example by assessing the degree to which between-tissue signals were driving the performance for the full set of 10 tissues in the pan-cancer analysis through the comparisons of the initial and tissue-standardized performance (**Figure 4C-D**).

Comment 3.9. Page 12: Line 217: Please clarify what between-tissue signal means. Does it mean difference in gene expression or drug sensitivities between brain and pancreatic cancer cell lines?

It's both: the between-tissue signal refers to the differences in both drug response and molecular characteristics between tissues. We have revised the text (**lines 244-246**): *[…] the two tissues are different in both drug response and molecular profiles (Figure 1C, E) and the performance across the two tissues in this example is driven by between-tissue differences.*

Comment 3.10. Page 15: Line 239-241: It is an important research topic to identify predictive genetic and transcriptomic markers that determines the drug sensitivity of MEKi. It may [not] (sic) be a bad idea to elaborate about molecular features that were finally included in each of pan-cancer regression models and how many common features were across the models.

We agree that the biomarkers selected by the models would be a useful resource to the community. We have generated four supplemental datasets – one for each regularized model – with biomarker information. This information includes mean importance and the amount of within- and between-tissue MEKi response explained for all features. The new Supplemental Datasets are references on **lines 279-281** in the revised Results.

Additionally, we generated a **new Supplemental Table 2** that indicates the counts of overlapping top 50 biomarkers between the four models. These results are described in the revised Results section (**lines 281-283**)

Comment 3.11. Page 15: Line 245: This is a very interesting observation. During down sampling of the pan-cancer data, did you randomly sample cell lines or

For the down-sampling, we randomly sampled without replacement from all available training cell lines. Tissues that are more prevalent in the full datasets tend to have more cell lines sampled, as we did not do weighted sampling.

Comment 3.12. Page 17: Line 262-265: I don't think only ovarian cancer cell line will be benefited from a larger pan-cancer data training set. As the sample size of a training set increases, stomach cancer cell lines also seem to show persistently steep increment in tissue-specific prediction performance.

We have revised the wording to indicate that ovarian cancers are “particularly” well-suited to larger sample sizes (**line 321**), as ovarian cancer was the only type with no inflection point of diminishing returns. This updated wording is less exclusive to other cancer types, such as stomach, which also showed increasing, albeit diminishing, performance returns with additional cell lines.
