# Supplementary material for: Impact of between-tissue differences on pan-cancer predictions of drug sensitivity
Source: PLoS Comput Biol. 2021 Feb 25;17(2):e1008720. doi: 10.1371/journal.pcbi.1008720 (PMC7906305; doi:10.1371/journal.pcbi.1008720)

Comment 2.1.3. The calculation of AUC for regression task is confusing and questionable. Please report in the figures and text CI instead of AUC, since it works both for regression and classification tasks. Also, do not use the term auROC, rather AUC or AUC-ROC, as those are standards in the field; the use of non-standard terms or calculations of AUC will make it difficult for others to reproduce the results.

We have adopted the Reviewer's suggestion by replacing the panel for AUC-ROC in **Figure 2D** with the one for Concordance, and modified the text accordingly (in Results, lines 188-215). We have updated **Table S1** to include ρ and AUC-ROC, and removed r as it was largely redundant with r^2. We replaced references to ‘auROC’ in the revised manuscript with ‘AUC-ROC’ (lines 215 and 593).

Figure 2D, <u>initial</u>:

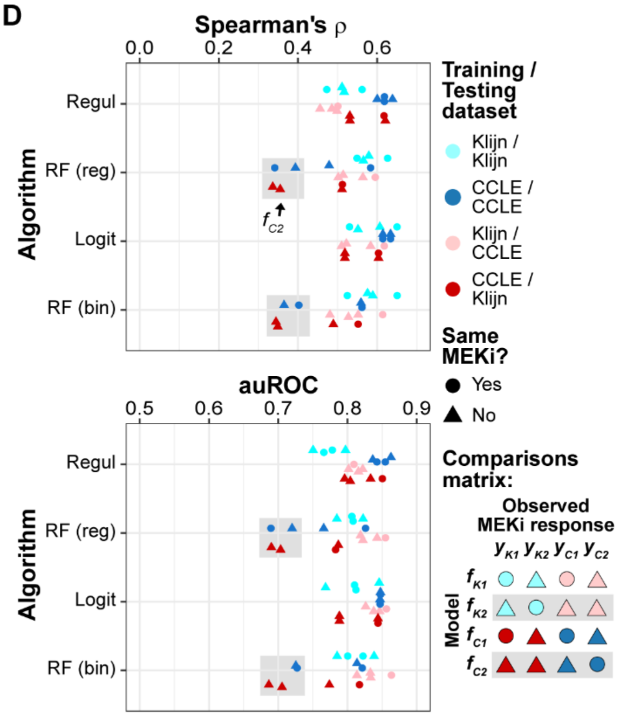

Figure 2D, <u>revised</u>:

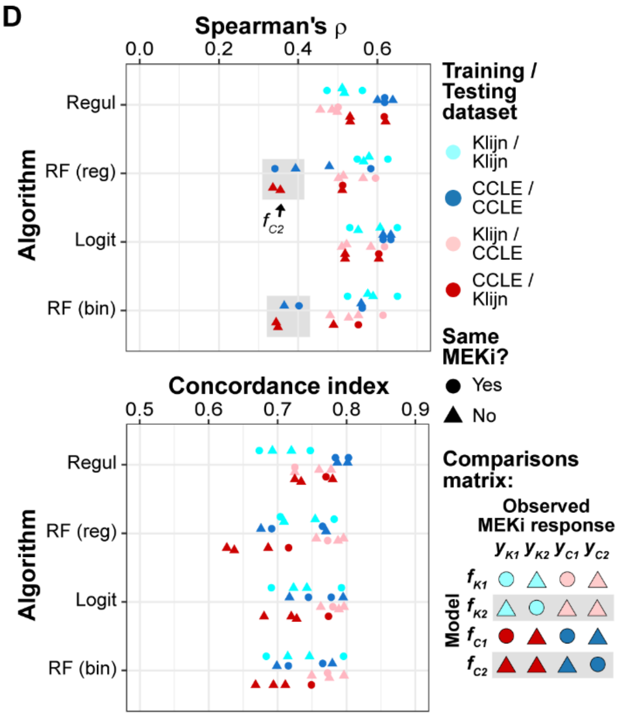

Comment 2.2.13: The authors showed similar prediction performances from the logistic regression with LASSO feature selection and logistic LASSO. This might be sufficient for the purpose of drug response prediction only. However, the authors should also make a comparison with respect to feature selection and effect estimation, as these are other important factors for the drug response modelling.

Our work is focused on between-group and within-group differences in contributing to overall performance. When discussing biomarkers and their effect sizes, we have only used results from regularized regression, not logistic regression. Therefore, the finer comparisons of the latter - two different ways of running logistic regression - would not affect the results for between- and

within-group comparisons as presented in **Figure 4F-H**, **Figure S5**, **Table S2**, and **Supplemental Datasets 1-4**.

We agree with the Reviewer that those interested in exploring details would want to compare feature selection and effect estimates between logistic regression with LASSO feature selection and logistic LASSO. To facilitate these comparisons, we have made the codes and procedures available.

Comment 2.2.14: It remained unclear from the response whether the authors added the 11 binary features for tissue-of-origin as mandatory variables in the regularised regression, i.e., without feature selection for the tissue features. If not, this should be done for proper evaluation of their importance. An additional try is to regress the drug responses on the tissue-label features only, without including any genomic data.

The 11 binary features are added but not as mandatory variables. However, the two models trained on CCLE data (f_{C1} and f_{C2}) utilized the ridge regression algorithm (α=0 was selected based on cross-validation), and nearly all features – including the 11 tissue features – were included in the models. The two models trained on Klijn 2015 data (f_{K1} and f_{K2}) utilized the elastic net algorithm (α=0.1 was optimal based on cross-validation), resulting in feature exclusion. As a result, in the f_{K1} and f_{K2} models, 9 of 11 tissue labels were not selected. As stated in the previous response, ".. *tissue type is aligned with a subset of molecular features, thus adding tissue label did not add much new information.*" Thus, it is not surprising that tissue labels are not typically selected when $\alpha>0$, as there are many more molecular features available for selecting the best features.

We also followed the Reviewer's second suggestion by running a regularized regression model that considers only Tissue Label as the feature (coded as 11 binary features). We then compared the performance of this tissue-only model with those of (1) the initial pan-cancer model, which considered all molecular features, and (2) the tissue-standardized model, which centered the observed and predicted MEKi response within tissues. The predictions based on the tissue-only model were worse than pan-cancer predictions (U test, $p < 4\text{x}10^{-4}$; revised **Figure 4C**, copied below), and similar to the tissue-standardized predictions (U test, p = 1; revised **Figure 4C**). This is not surprising as we have shown tissue-specific patterns for both the drug response (Fig.1C) and the molecular features (Fig.1E). We revised Results and Discussion (lines 261-268) to include this new analysis, and added the tissue-only model to the three-way comparison in the revised **Figure 4C**. The new 4C replaced the initial 4C-D (copied below) which did not include the tissue-only models.

Figure 4C, <u>revised</u>:

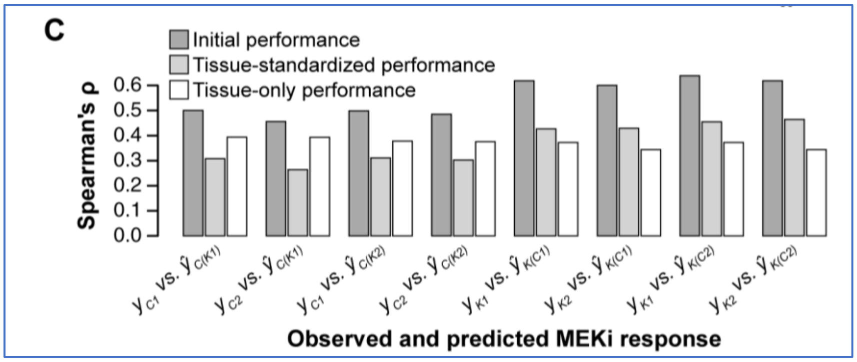

Figure 4C-D, <u>initial</u>:

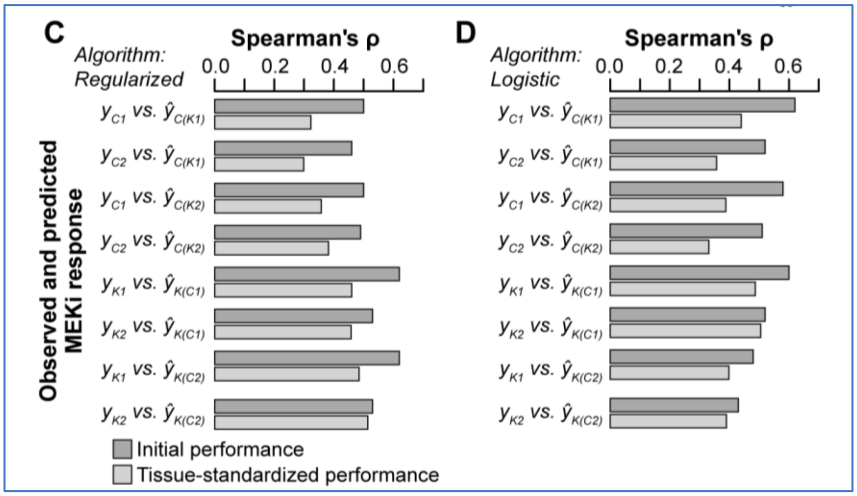

Supplement: Attachment — Submitted filename: response_to_reviewers_Dec21.pdf [file pcbi.1008720.s007.pdf]
